# Supplementary material for: Diffusing wave paradox of phototactic particles in traveling light pulses
Source: Nat Commun. 2019 Jun 7;10:2495. doi: 10.1038/s41467-019-10535-z (PMC6555803; doi:10.1038/s41467-019-10535-z)
Supplement: Supplementary file 2 — Description of Additional Supplementary Files [file 41467_2019_10535_MOESM2_ESM.pdf]

Description of Additional Supplementary Files

File Name: Supplementary Movie 1

Description: Positive phototaxis of active particles (σ=3.25 µm) in a one-dimensional static light gradient = 0.007 µW µm−3 . Movies are accelerated by factor 3.

File Name: Supplementary Movie 2

Description: Response of active particles (σ=3.25 µm) to a periodic train of pulses with velocity u = 2 µm/s (u/v_p^{max} =1), width w =4σ and T/(1/Dr) = 0.1. To illustrate the traveling train pulses, they are shown schematically on the upper part of the video. Note that particles with different orientations are transported in opposite directions. Movies are accelerated by factor 2.

File Name: Supplementary Movie 3

Description: Response of active particles (σ=3.25 µm) to a periodic train of pulses with velocity u= 2 µm/s (u/v_p^{max} =1), width w =4σ and T/(1/Dr) = 2. Note that the acceleration of the movie is slowed down when the particle is interacting with a pulse (see upper right corner) to enhance the visibility of the particle response to a running pulse.

File Name: Supplementary Movie 4

Description: Sorting of a mixture of small (σ=3.25 µm) and large (σ=4.9 µm) active particles to a periodic train of pulses with velocity u= 2 µm/s (u/v_p^{max} =1), width w =4σ and T = 5s. Movies are accelerated by factor 2.
